# MXene-Functionalized Light-Induced Antimicrobial and Waterproof Polyacrylate Coating for Cementitious Materials Protection

**DOI:** 10.3390/polym15092076

**Published:** 2023-04-27

**Authors:** Hongping Zhang, Pengfei Tang, Youhong Tang, Kun Yang, Qingyuan Wang

**Affiliations:** 1 School of Mechanical Engineering, Institute for Advanced Study, Chengdu University, Chengdu 610106, China; 2 Failure Mechanics and Engineering Disaster Prevention and Mitigation Key Laboratory of Sichuan Province, College of Architecture and Environment, Sichuan University, Chengdu 610065, China; 3 Institute for Nanoscale Science and Technology, College of Science and Engineering, Flinders University, Adelaide, SA 5042, Australia

**Keywords:** protection coating, Ti_3_C_2_ MXene, waterproof, antimicrobial, cement-based materials

## Abstract

The penetration of external stimuli (microorganisms, ions, etc.) following to the pore is the key reason for the deterioration of cement and concrete structures. Although the traditional methods such as improving the chemical composition of cement and concrete materials can delay the erosion rate, the inevitable pore structure still makes its deterioration a challenge. Based on this, we reported a protective coating for cementitious materials based on phenol and Ti_3_C_2_ MXene-modified polyacrylate (MXene-PG/PA). The introduction of phenols enhanced the waterproof properties of polyacrylate by increasing the interaction among molecular chains. Moreover, the introduction of Ti_3_C_2_ MXene also endows the MXene-PG/PA coating with good light-induced antimicrobial properties. Beneficial to these designs, the MXene-PG/PA coating exhibited good waterproof properties (the water absorption ratio in seawater decreased by 58.2%) and antimicrobial properties (inhibition of *E. coli* and *S. epidermidis* activity under light). These results not only confirm that the MXene-PG/PA coating is a potential candidate of protective coating for cement-based materials, but also provide a new strategy for the design of multifunctional protective coatings.

## 1. Introduction

Due to the presence of the pores and capillary network structures in concrete materials, external stimuli usually enter the interior of cement and concrete materials through these structures, and finally result in the deterioration of the structure and performance [1]. For example, the concrete structure and the infrastructure often suffers degradation because of water ingress. Subsequently, problems such as rebar corrosion eventually arise due to the infiltration of chlorides, acids, and other corrosive chemicals [2]. The acid corrosion produced by the activity of microorganisms is also one of the factors leading to the deterioration of the performance of cement-based materials [3]. Generally, the physiological activities of microorganisms will produce a large amount of CO_2_ and organic acids (including succinic acid, acetic acid, and lactic acid [4,5]) substances. As for Portland cement and its alkaline by-products, corrosion reactions usually occur when the pH is below 12.5. This eventually leads to severe biological deterioration of the physical and chemical properties of the material. In addition to the dissolution of hydration products caused by acid corrosion, the CO_2_ produced by microbial metabolism will also aggravate the carbonation of concrete, resulting in a decrease in its performance [6]. However, traditional protection technologies lack effective resistance to these corrosion factors. This ultimately poses challenges to the reliability of cement and concrete materials. Therefore, there is an urgent need to reduce the environmental corrosion of concrete structures and improve their durability through appropriate protective treatment technologies.

To improve the durability of concrete, especially for the ions and microorganisms in water, methods including the introduction of functional materials (TiO_2_ [7], ZnO [8], graphene-based materials [9], carbon nanotubes [10], etc.) to concrete and modified by protective coatings [11,12] have been proposed. For example, an antibacterial concrete was constructed by introducing ZnO particles in the concrete, which achieved the inactivation of bacteria and fungi [8]. José et al. [13] evaluated the addition of crystalline hydrophilic additives to concrete to form new phases (by adjusting the hydration reaction of cement), and, finally, the water permeability of concrete decreased by 50%. These studies have evidenced that the introduction of functional materials can effectively improve the protective performance of concrete. However, the introduction of different functional materials impacts parameters such as setting time and mechanical properties of cement-based materials [14,15]. In contrast, surface protective coatings modification is a better choice for concrete protection. Unfortunately, due to the lack of functional phases (the waterproofing additive and antibacterial additives), it is difficult for traditional protective coatings to protect against special corrosion forms such as microorganisms. Therefore, it is urgent to enrich the functions of protective coatings through reasonable design.

**Figure 1:**
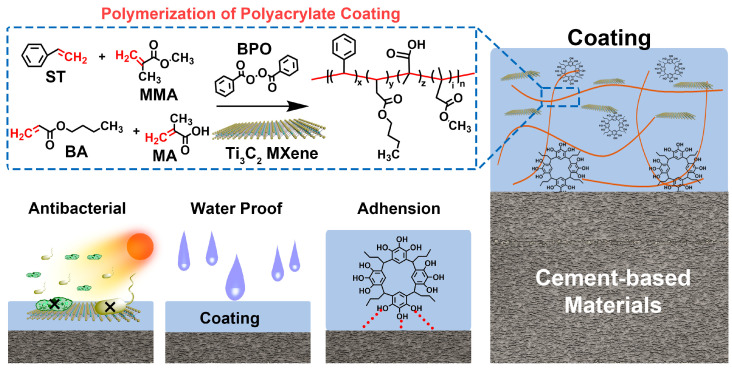
Design and fabrication process of the waterproof and anti-microorganism coating.

With the advancement of composite technology, many functional coatings have been developed by introducing functional phases in the coating matrix [16]. One such development is the photothermal conversion coatings, which convert the captured light energy into heat through the photothermal conversion effect and generate high temperatures to inactivate microorganisms on the surface to achieve anti-microbial fouling [17]. These achievements also offer new strategies for designing protective coatings for cement and concrete materials. In this study, we have developed a coating that offers dual protection against ionization in water and microbial corrosion for cement-based materials (Figure 1). To achieve this goal, we compounded phenol (polypyrogallol) into the PA coating, which enhances the adhesion and waterproof properties of the coating by improving the interaction between molecular chains. Additionally, we incorporated Ti_3_C_2_ MXene as a functional phase to the PG/PA coating, providing excellent photothermal conversion properties that effectively inhibit bacterial growth in the presence of light.

## 2. Materials and Methods

### 2.1. Materials

The styrene (ST), methyl methacrylate (MMA), butyl acrylate (BA), methacrylic acid (MA), benzoyl peroxide (BPO), and xylene were purchased from Chengdu Jinshan chemical reagent Co., Ltd., Chengdu, China. Dopamine hydrochloride (DA) and tannic acid (TA) were purchased from Shanghai Aladdin Biochemical Technology Co., Ltd., Shanghai, China. Ti_3_C_2_ MXene dispersion (100 mg/mL) were obtained from Jilin Yiyi Technology Vo., Ltd., Jilin, China.

### 2.2. The Fabrication of the Composite Coating

#### 2.2.1. The Fabrication of Polypyrogallol (PG)

Polypyrogallol was prepared according to the previous study [18]. Briefly, 7.21 mL (0.08 mol) of n-butyraldehyde and 10 g (0.08 mol) of pyrogallol were dissolved in 40 mL of methanol. 3.5 mL of concentrated hydrochloric acid (36 wt%) was then added to the homogeneously mixed solution. After the hydrochloric acid was added dropwise, the mixed solution was refluxed at 110 °C for 24 h. After the reaction was completed, the solution was cooled to room temperature and filtered to obtain a white precipitate after cooling. The precipitate was washed with methanol and vacuum-dried (or freeze-dried) to obtain polypyrogallol powder.

#### 2.2.2. The Fabrication of the Phenol-Modified Polyacrylate

A solution was prepared by adding styrene (13 g), methyl methacrylate (38 g), butyl acrylate (27 g), and methacrylic acid (2 g) to xylene (80 g) at 110 °C. After uniform stirring, the toluene solution containing benzoyl peroxide was slowly added dropwise to the above solution. After heat preservation for 30 min, the phenol-modified polyacrylate sol was obtained by adding the phenol (dopamine, pyrogallol, and tannic acid) solutions to the polyacrylate sol (the total concentrations of phenol are 0.5 mg/mL, 1 mg/mL, 2 mg/mL, 5 mg/mL, and 10 mg/mL, respectively). The coatings obtained by dopamine, tannic acid, and pyrogallol modified PA were defined as DA/PA, TA/PA, and PG/PA, respectively.

#### 2.2.3. The Fabrication of the Ti_3_C_2_ MXene Modified PG/PA (MXene-PG/PA)

Prior to the experiment, the water in Ti_3_C_2_ MXene dispersion was replaced by DMSO through high-speed centrifugation (at 10,000 rpm, 5 min). The Ti_3_C_2_ MXene dispersion was then mixed with the PG/PA sol (phenol concentration 2 mg/mL). Finally, the Ti_3_C_2_ MXene modified PG/PA coating was obtained after stirring and the polymerization reaction. The obtained MXene-PG/PA coating with 0.025 wt%, 0.05 wt%, 0.075 wt%, and 0.1 wt% Ti_3_C_2_ MXene were defined as 0.025 MXene-PG/PA, 0.05 MXene-PG/PA, 0.075 MXene-PG/PA, and 0.1 MXene-PG/PA.

### 2.3. Characterizations

The microstructure of the coating was analyzed by using a scanning electron microscope (SEM, Ultra 55, Carl Zeiss, Oberkochen, Germany). The viscosity changes of PG/PA coatings at various temperatures (5–45 °C) and frequencies (1–10 Hz) were examined by using a rotational rheometer (HAAKE RS1, Freden, Germany).

### 2.4. Swelling Ratio

The swelling ratio of the coating was tested according to the previous study [18]. Briefly, the coating was dried at 80 °C to a constant weight and the mass (*m*_0_) was recorded, and then it was immersed in deionized water. The sample was taken at a specific time and the mass (*m*_1_) was recorded. The swelling ratio is calculated by the following equation.

$$\mathrm{Swelling}\;\mathrm{ratio}=\frac{m_{1}-m_{0}}{m_{0}}$$

### 2.5. Adhesion to Substrates

The adhesion of PA coatings on cement samples was tested according to ASTM D3359-17 standard [19,20]. Before the test, the coating precursor (sol) was brushed on the surface of the cement first. The coating was then obtained after the polymerization reactions. The crosshatch tests were performed for the coatings on the cement sample surface. The adhesion grade was evaluated according to the crosshatches.

### 2.6. Waterproof Performance

The waterproof performance was evaluated by the water absorption ratio according to the previous study [21]. Before the test, the coating precursor (sol) was brushed on the surface of the cement block, and the coating was obtained after the polymerization reactions. The cement blocks were then placed in an oven at 105 °C for 72 h. Next, the sample was removed from the oven and cooled to room temperature. Then, the cement blocks were weighed (*m*_0_) and immersed in water (deionized water and simulated seawater, respectively). After a certain time, the sample was removed and the surface was wiped to record the weight (*m_t_*) again. The water absorption ratio was calculated by the following equation:

$$\mathrm{Water}\;\mathrm{absoption}\;\mathrm{ratio}=\frac{m_{t}-m_{0}}{m_{0}}$$

### 2.7. Antimicrobial Test

The anti-microorganism properties of the coatings were evaluated based on previous studies by analyzing their ability to inhibit bacteria (Escherichia coli and Staphylococcus epidermidis) [22]. In short, the MXene-PG/PA coating was placed into a 24-well plate, and 100 μL of bacterial suspension (*E. coli*, and *S. epidermidis*) was added onto the coating surface. Next, the coculture systems were transferred to a 37 °C environment for cultivation. The system was then exposed to light treatment (50 W, 1 h). Next, 900 μL of agar protein medium was added and continued to be cultured at 37 °C for 12 h. Finally, the antimicrobial properties of the coatings under light were evaluated by the optical density method and plate count method, respectively. The microscopic morphology of the bacteria was observed through SEM (Ultra 55, Carl Zeiss, Oberkochen, Germany). Prior to observation, the specimens were fixed with a glutaraldehyde solution (2.5 wt.%) and subsequently freeze-dried to acquire the samples.

## 3. Results and Discussion

### 3.1. The Structure and the Performance of the PA Coatings

**Figure 2:**
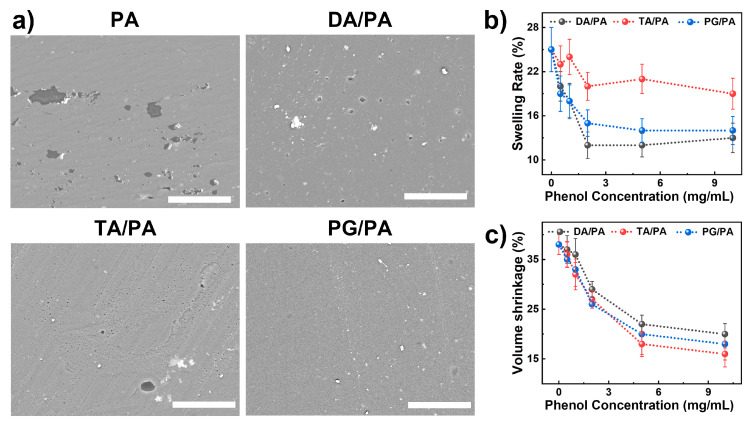
(**a**) Microscopy, (**b**) swelling ratio, and (**c**) volume shrinkage of the polyacrylate (PA) coating modified by various phenols (dopamine, tannic acid, and polypyrogallol; 2 mg/mL). The scale bar is 50 μm.

Microstructure of the protective coating is essential for achieving cement-based protection, and a complete coating can effectively isolate external stimuli [23]. The SEM image (Figure 2a) shows that the pure PA coating is smooth but still has many micro defects. Interestingly, the introduction of phenols can effectively reduce these defects, where DA/PA, TA/PA, and PG/PA coatings show smooth surfaces (Figure 2a). It is worth noting that an increase in phenol concentration results in visible pores on the surface of the composite coating (see Figure S1). Conversely, lower phenol concentrations lead to a denser coating without observable pores. At lower concentrations of PG, the decrease in defects in the PG/PA coating may be linked to the reaction rate during the polymerization of the PA coating. During the fabrication of PA coatings, the initiation of free radicals formed by the decomposition of BPO is crucial to the polymerization of the coating. However, the surface of phenolic substances is rich in reducing catechol functional groups that can capture and quench free radicals during polymerization, slowing down the polymerization rate of PA coatings. A slower polymerization rate results in a more uniform structure during polymerization, leading to fewer defects in the PA coating. However, as the concentration of phenol increased, the excess trapped free radicals had a negative impact on the polymerization reaction of the PA coating, resulting in a poor degree of polymerization. Consequently, during the drying process, as the solvent evaporates, larger defects are formed.

In addition to the microscopic morphology, Figure 2b,c also demonstrate the swelling rate and volume shrinkage rate of the PA-based coating after phenol introduction. The swelling rate and volume shrinkage of the PA coating are related to the content of phenols. Among them, the swelling rate decreased rapidly with the increase in phenol concentration and remained almost constant when the phenol concentration was higher than 2 mg/mL. Notably, the DA/PA coating exhibited the lowest swelling rate of 12.8% at a phenol concentration of 2 mg/mL, compared to TA/PA (20.4%) and PG/PA (15.3%). The difference in the swelling rate may primarily be attributed to the molecular structure of phenol. In comparison to TA and PG, DA had the smallest structural unit, which facilitates better contact with the matrix material, enhances the interaction between molecular chains, and ultimately results in the lowest swelling rate. As for the volume shrinkage, a similar rule can be observed, that is, the volume shrinkage of PA coatings decreases with the increase in phenol concentration. Furthermore, due to the smaller structural unit and the robust interaction between molecular chains, DA/PA demonstrates a higher volume shrinkage rate compared to other blends. At a concentration of 5 mg/mL of phenolic substances, the volume shrinkage rates for DA/PA, PG/PA, and TA/PA were 22.4%, 20.3%, and 18.1%, respectively. The swelling ratio and volume shrinkage ratio results indicate that the addition of phenol can significantly enhance the performance of the PA coating, particularly for PG.

The adhesion of coating to a substrate is also a critical parameter. To evaluate adhesion of the PA coating to cement substrates, the crosshatch test was performed on the PA coating of the cement surfaces. Figure S2 shows the diagrammatic sketch of the crosshatch method of coating with various concentrations of phenols, and the evaluated adhesion grades are shown in Table 1. As expected, the introduction of the phenol enhanced the adhesion ability of the PA coating, where PG/PA, DA/PA, and TA/PA showed the highest adhesion grade (5A) at the phenol concentration of 2 mg/mL. However, the adhesion grade decreased when the phenol concentration was higher than 2 mg/mL. The increase in the pore structure should be responsible for the decrease in the adhesion grade, where the pore structure increased with the increase in the phenol content (Figure S1). Due to the superior performance in terms of swelling, volume shrinkage, and adhesion properties, the PG/PA coating (2 mg/mL PG) was selected for further research.

**Figure 3:**
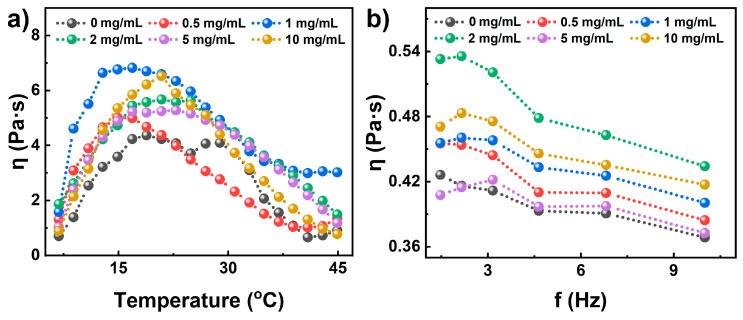
The viscosity of the solution precursor of PG/PA coating at various (**a**) temperatures and (**b**) frequencies.

The viscosity of the solution precursor is also a critical parameter for coating [17]. To explore the change in viscosity of the solution precursor with PG concentrations, the viscosity of PG/PA solution precursor at different temperatures (5–45 °C) and frequencies (1–10 Hz) was studied. It can be found from the viscosity versus temperature curves of the solution precursor of PA-based coatings (Figure 3a) that the temperature is a key parameter affecting the solution precursor of PA coatings. The viscosity of the solution precursor for PA coating increases and then decreases with rising temperature. Additionally, the viscosity of the solution precursor for PG/PA coatings follows a similar law with temperature. Nevertheless, the viscosity of the solution precursor of PG/PA coating is linked to the concentration of PG. It increases at low concentrations (≤1 mg/mL) and decreases at high concentrations (>1 mg/mL). The change in precursor viscosity with temperature may be attributed to the interaction between phenol and PA coating molecular chains, as well as the movement of PA molecular chains. At lower temperatures, the interaction between phenol and PA molecular chains intensifies, leading to an increase in viscosity with rising temperature. However, as the temperature continues to rise, the degrees of freedom for molecular chains within the PA also increases, causing a decrease in viscosity.

In addition to the viscosity change with the temperature, Figure 3b shows the change in viscosity of the solution precursor versus frequencies. It is worth noting that the viscosity of the solution precursor decreased as the frequency increased, whereas the solution precursor of the PA coating decreased from 0.43 Pa·s (0.15 Hz) to 0.37 Pa·s (10 Hz). In addition, the viscosity of the solution precursor increased at low concentrations (≤2 mg/mL) and decreased at high concentrations (>2 mg/mL). The difference in viscosity may be attributed to the interactions between PG and polyacrylate resin. At low concentrations, PG increases the interactions among polyacrylate molecular chains by forming hydrogen bonds and other physical effects, while at high concentrations, too much PG inhibits the combinations among polyacrylate molecular chains. An appropriate viscosity makes PG/PA coating a good candidate for the protection of cementitious material.

**Figure 4:**
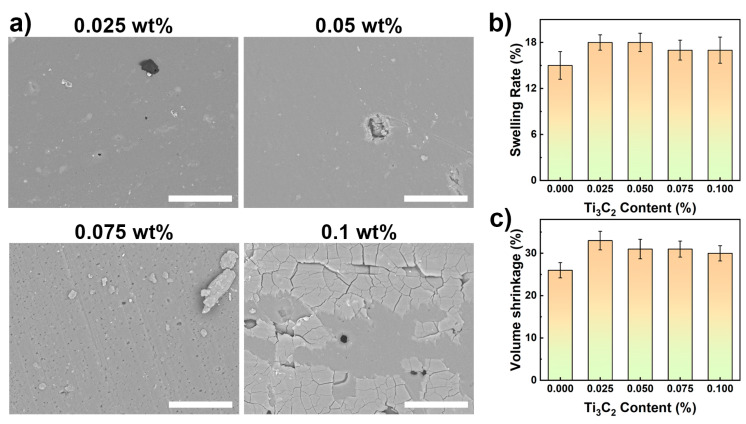
(**a**) Microscopy (**b**) swelling ratio, and (**c**) volume shrinkage of the Ti_3_C_2_ MXene modified PG/PA (PG: 2 mg/mL) coatings, the scale bar is 50 μm.

To improve the antimicrobial performance of PG/PA coatings, Ti_3_C_2_ MXene with excellent photothermal conversion performance was introduced into PG/PA coatings [24]. Figure 4a exhibits the microscopy of MXene-PG/PA coatings. It can be found that the addition of a small amount (0.25 wt%) of Ti_3_C_2_ MXene does not affect the surface structure of the PG/PA coating. However, excessive incorporation of Ti_3_C_2_ MXene caused the appearance of some void structures and agglomeration on the coating surface. This phenomenon can be explained by the acceleration of Ti_3_C_2_ MXene for the coating curing process, as previous studies have also demonstrated that Ti_3_C_2_ MXene can catalyze polymerization reaction through oxidation reaction equilibrium [22].

Furthermore, Figure 4b investigates the alteration of the swelling ratio of the composite coating after incorporating Ti_3_C_2_ MXene. As depicted in the figure, the swelling rate of the PG/PA coating is 15.3%, and when the proportion of Ti_3_C_2_ MXene increased to 0.025%, 0.05%, 0.075%, and 0.1%, the swelling ratios were increased to 18.5%, 18.3%, 17.6%, and 17.2%, respectively. The variation of volume shrinkage with respect to the Ti_3_C_2_ MXene content is demonstrated in Figure 4c. As the Ti_3_C_2_ MXene content increased from 0% to 0.025%, 0.05%, 0.075%, and 0.1%, its volume shrinkage increased from 26% to 33.2%, 31.6%, 31.3%, and 30.3%, respectively. These findings suggest that Ti_3_C_2_ MXene can be introduced as a functional phase without significantly affecting the performance of the PA coatings.

### 3.2. Waterproof Performance of the Coatings

**Figure 5:**
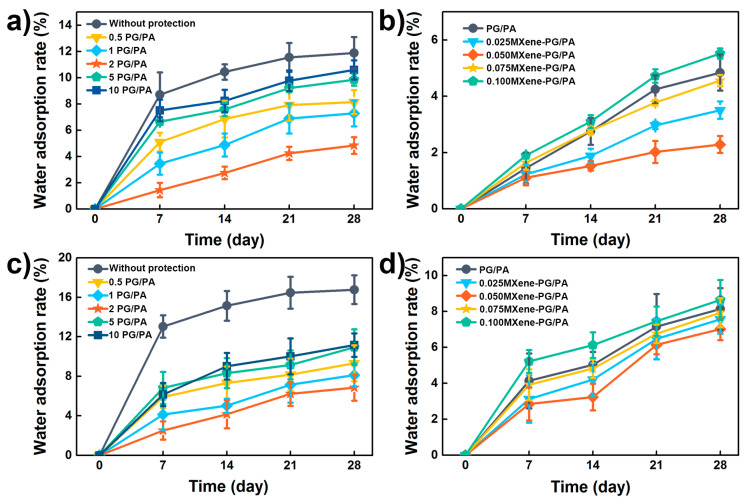
Water absorption of (**a**) PG/PA coating with various PG content and (**b**) MXene-PG/PA coating with various MXene content in deionized water. (**c**,**d**) The water absorption of PG/PA coating and MXene-PG/PA coating in seawater.

Generally, water absorption is a key parameter for the waterproofing of coatings [19,20]. Therefore, the water absorption behavior of coatings (including PG/PA coating and MXene-PG/PA coating) in deionized water and seawater were investigated, respectively. Prior to testing, the coating was brushed on the surface of the cement test block, and its waterproof properties were assessed by measuring the mass variation following immersion. Figure 5a shows the water absorption of the pure cement block and the cement block with PA-based protective coatings in water. Due to the lack of a protective coating, the pure cement block showed poor waterproof performance, whereas the water absorption of pure cement block increased rapidly to 8.9% within 7 days and reached 12.1% at 28 days. However, the waterproof performance increased with the introduction of PG/PA protected coatings (As is shown in Figure 5a). With the increase in the PG concentration, the water absorption rate first decreased (reached the minimum at the concentration of 2 mg/mL) and then increased. At low PG concentrations (<2 mg/mL), the dense surface structure enhanced by PG should be the key to reduce its water absorption rate. As the PG concentration increased, the porous structure of the coating facilitated the transport of water, which eventually led to an increase in its water absorption rate.

Except for the PG/PA coatings, the water absorption ratio of the MXene-PG/PA coating was also evaluated (as shown in Figure 5b). The water absorption of the cement blocks with PG/PA coating is 4.6% at 28 days. With the addition of 0.025 wt% and 0.05 wt% Ti_3_C_2_ MXene, the water absorption ratio decreased to 3.7%, and 2.1%, respectively. This can be explained by the dense structure of the coatings. However, the formed pore structure and the caking structure at high Ti_3_C_2_ MXene contents facilitate the water transportation, and the water absorption ratio therefore increased to 4.2% and 5.5% when the Ti_3_C_2_ MXene content increased to 0.075 wt%, and 0.1 wt%. All these results confirmed the good waterproof performance of MXene-PG/PA coatings.

Generally, cement-based materials are also served in the marine environment and suffer corrosion from the seawater ions (Especially Cl^−^, CO_3_^2−^, SO_4_^2−^). Therefore, the waterproof performance in seawater was investigated. It is worth noting that the water absorption ratio of cement-based materials in seawater shows similar behavior to that of deionized water. The 2-PG/PA coating shows the lowest water absorption ratio of 7.5% at 28 days (as shown in Figure 5c). Moreover, the MXene-PG/PA coating with 0.05 wt% Ti_3_C_2_ MXene shows the lowest water absorption ratio of 6.8% (at 28 days). The disparity lies in the fact that the cement samples displayed higher water absorption when placed in seawater as opposed to deionized water. In short, the good water absorption ratio confirmed the MXene-PG/PA coating can serve as a good candidate for the protection of cement materials in the marine environments.

### 3.3. Anti-Microorganism Performance of the Coatings

**Figure 6:**
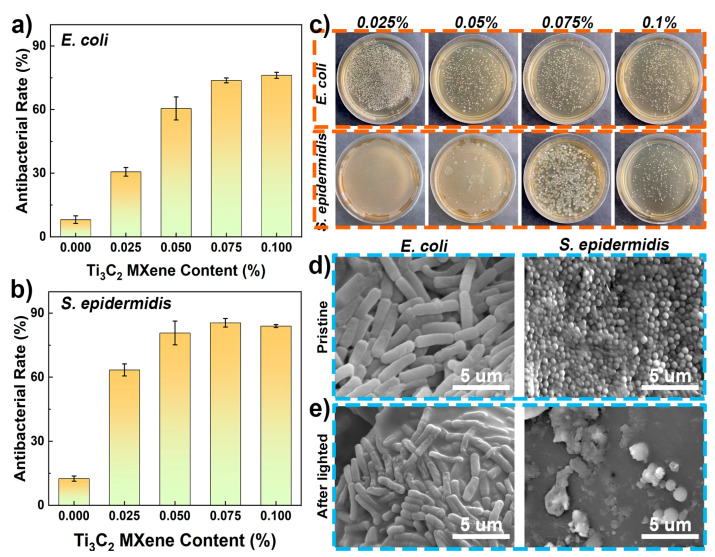
Light-induced anti-bacterial performance of the PA-based coating. (**a**,**b**) The anti-bacterial ratio for Escherichia coli (*E. coli*) and Staphylococcus epidermidis (*S. epidermidis*). (**c**) The photographs of agar plates of colonies of *E. coli* and *S. epidermidis*. The morphology of the bacteria (*E. coli*, and *S. epidermidis*) on the (**d**) pristine coating and (**e**) the coating after light treatment.

The antimicrobial performance of the coating under light was evaluated by studying the antimicrobial properties of the coatings. As shown in Figure 6a,b, due to the lack of the photothermal conversion agents (Ti_3_C_2_ MXene), the pure PG/PA coating shows a lower anti-microbial ability, where the anti-bacterial ratios are 10% and 12% for the *E. coli* and *S. epidermidis*. Of note, the antibacterial performance of the MXene-PG/PA coating depends on the content of Ti_3_C_2_ MXene. The antibacterial ratio increased with the increase in the Ti_3_C_2_ MXene content and reached a maximum of 75% (*E. coli*) and 80% (*S. epidermidis*) when the Ti_3_C_2_ MXene content was 0.075 wt%. Afterward, the anti-bacteria ratio remains essentially constant. The photographs of agar plates of colonies of *E. coli* and *S. epidermidis* also confirmed this result, where the number of colonies decreased rapidly when the Ti_3_C_2_ MXene content exceeded 0.075 wt%. More information about the morphology of the bacteria (*E. coli* and *S. epidermidis*) were shown in Figure 6d,e. Upon observation, *Escherichia coli* and *Staphylococcus epidermidis* exhibited typical structures prior to light exposure. After light treatment, both bacterial strains demonstrated the rupture of their cell membranes. These findings suggest that the MXene-PG/PA coating displays inhibitory properties against bacteria following light treatment. The good anti-bacterial performance of the composite coating is primarily attributed to the excellent photo-thermal conversion properties of Ti_3_C_2_ MXene. Prior research has demonstrated that Ti_3_C_2_ MXene exhibits remarkable light absorption capabilities along with a nearly 100% photo-thermal conversion efficiency. Consequently, the incorporation of Ti_3_C_2_ MXene contributes significantly towards enhancing the light-to-heat conversion properties of the composite coating. Upon irradiation with light, the photo-thermal conversion effect converts the light into thermal energy, effectively inhibiting bacterial growth. All these results have confirmed the MXene-PG/PA coating has a good anti-microorganism performance, which makes it a potential candidate for the protective coating of cement-based materials.

## 4. Conclusions

In summary, we demonstrated that Ti_3_C_2_ MXene and PG modified polyacrylate coatings can serve as protective coatings for cement-based materials. Compared to other phenols (dopamine and tannic acid), the PG modified PA coating has a smoother surface structure. At the same time, the PG-modified PA coating has good adhesion and waterproof properties. Moreover, the addition of a small amount of Ti_3_C_2_ MXene can also enhance the waterproof performance of the PG/PA coating. Meanwhile, due to the good photothermal conversion of Ti_3_C_2_ MXene, the composed PA coating also shows good antimicrobial properties for *E. coli* and *S. epidermidis*. These results not only prove that the introduction of PG and Ti_3_C_2_ MXene can improve the waterproof and antimicrobial properties of polyacrylate coatings, but they also provide a new strategy for designing multifunctional protective coatings.

## Figures and Tables

**Table 1:** Adhesion grades of different types of composite coatings.

	0.5 mg/mL	1 mg/mL	2 mg/mL	5 mg/mL	10 mg/mL
PG/PA	4A	4A	5A	3A	2A
DA/PA	4A	4A	5A	2A	2A
TA/PA	3A	3A	5A	4A	3A

## Data Availability

The authors declared that they have no conflicts of interest to this study. We declare that we do not have any commercial or associative interest that represents a conflict of interest in connection with the study submitted.

## Supplementary Materials

The following supporting information can be downloaded at: https://www.mdpi.com/article/10.3390/polym15092076/s1, Figure S1. The microscopy of the polyacrylate coating with various phenols (DA, TA, and PG) contents, the scale bar is 50 μm; Figure S2. the diagrammatic sketch of the crosshatch method of coating with various phenol concentrations.
