# Supplementary material for: MXene-Functionalized Light-Induced Antimicrobial and Waterproof Polyacrylate Coating for Cementitious Materials Protection
Source: Polymers (Basel). 2023 Apr 27;15(9):2076. doi: 10.3390/polym15092076 (PMC10180540; doi:10.3390/polym15092076)
Supplement: Supplementary file 1 [file polymers-15-02076-s001.zip › polymers-2275238-supplementary.pdf]

Supporting Information

MXene-Functionalized Light-Induced Antimicrobial and Waterproof Polyacrylate Coating for Cementitious Materials Protection

Hongping Zhang [1,*], Pengfei Tang [2], Youhong Tang [3,*], Kun Yang [1], Qingyuan Wang [1,2]

1 School of Mechanical Engineering, Institute for Advanced Study, Chengdu University, Chengdu 610106, China.

2 Failure Mechanics and Engineering Disaster Prevention and Mitigation Key Laboratory of Sichuan Province, College of Architecture and Environment, Sichuan University, Chengdu 610065, China.

3 Institute for Nanoscale Science and Technology, College of Science and Engineering, Flinders University, South Australia 5042, Australia

* Correspondence: zhanghongping@cdu.edu.cn or zhp1006@126.com (H.Z.); youhong.tang@flinders.edu.au (Y.T.)

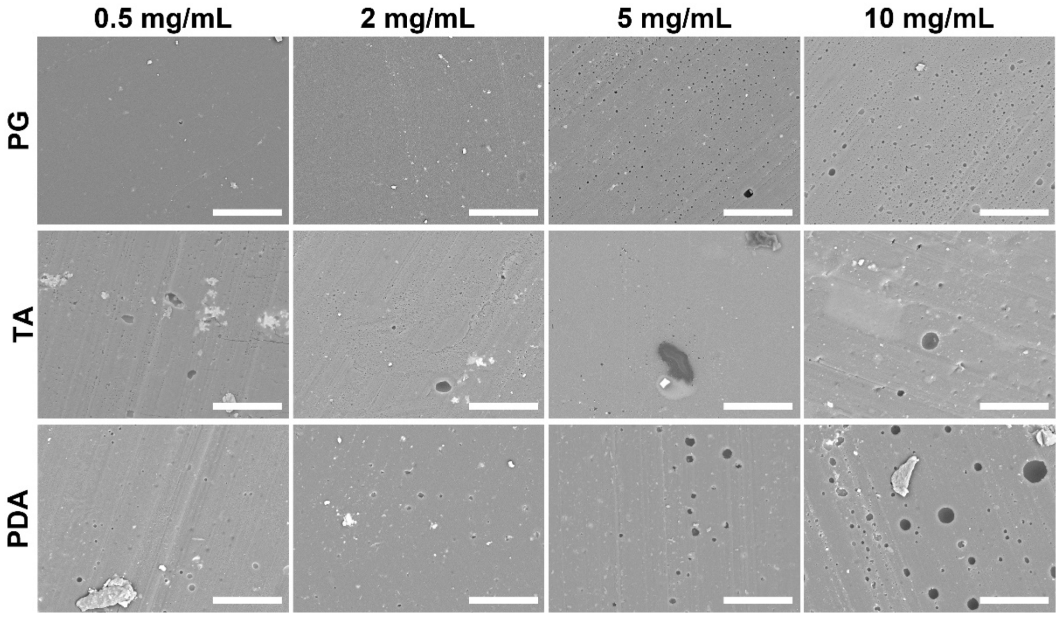

Figure S1. The microscopy of the polyacrylate coating with various phenols (DA, TA, and PG) contents, the scale bar is 50 μm.

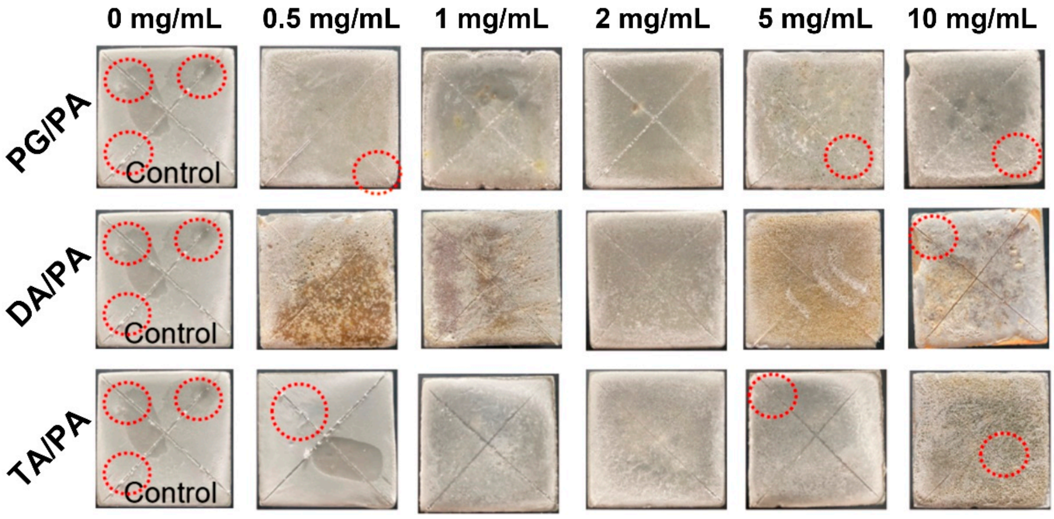

Figure S2. the diagrammatic sketch of the crosshatch method of coating with various phenol concentrations.
